# The Role of Geographical Differences and Discrimination in the Cognitive Health of Older Blacks

**DOI:** 10.1093/geroni/igaa057.3253

**Published:** 2020-12-16

**Authors:** Kimson Johnson, Ketlyne Sol, Briana Sprague, Tamara Cadet, Elizabeth Munoz, Noah Webster

**Affiliations:** 1 University of Michigan, Ann Arbor, Michigan, United States; 2 University of Pittsburgh, Pittsburgh, Pennsylvania, United States; 3 Simmons University, Boston, Massachusetts, United States; 4 The University of Texas at Austin, Austin, United States

## Abstract

Where you live in old age matters in the United States in terms of access to health care and potential exposure to discrimination. This is especially important for non-Hispanic Blacks. Everyday experiences of discrimination are known to “get under the skin” but to date few studies have examined if associations between discrimination and cognitive health differ by region and urbanicity. This study aims to fill this gap. Data are from the 2012 and 2014 waves of the Health and Retirement Study (HRS) (N =2,347). Non-Hispanic Blacks over age 51 with complete data on the Everyday Discrimination Scale in either 2012 or 2014 and cognition, residential location were included in the analysis. Regression models included covariates for age, gender, education and examined interactive effects of everyday discrimination, region, and urbanicity on episodic memory in a nationally representative sample of non-Hispanic older Black adults in the U.S. Descriptive analyses revealed that the majority lived in Southern regions and in non-urban areas. Respondents also reported experiencing discrimination a few times a year. Regression analyses indicated that experiencing more everyday discrimination was significantly associated with lower episodic memory when living in urban areas. Among Blacks, the discrimination-episodic memory link did not significantly vary across U.S. regional contexts. Findings highlight the importance of socio-environmental factors in shaping how stressful experiences such as everyday discrimination are linked to cognitive health in later life. Future research should focus on the development of focused upstream interventions that are responsive to racial disparities in cognitive health in urban areas.

